# Impact of intra-operative cell salvage on blood coagulation in high-bleeding-risk patients undergoing cardiac surgery with cardiopulmonary bypass: a prospective randomized and controlled trial

**DOI:** 10.1186/s12967-016-0986-6

**Published:** 2016-07-29

**Authors:** Sheliang Shen, Jun Zhang, Wenyuan Wang, Jiayin Zheng, Yihong Xie

**Affiliations:** 1 Department of Anesthesiology, Zhejiang Provincial People’s Hospital, Hangzhou, Zhejiang China; 2 Department of Orthopedics, Zhejiang Provincial People’s Hospital, Hangzhou, Zhejiang China; 3 Department of Probability and Statistics, School of Mathematical Sciences, Beijing University, Beijing, China; 4 Department of Cardiothoracic Surgery, Zhejiang Provincial People’s Hospital, Hangzhou, Zhejiang China

**Keywords:** Cell salvage, Cardiac surgery, High-risk, Bleeding, Coagulation

## Abstract

**Background:**

Intra-operative cell salvage (CS) was reported to have no impairment on blood coagulation in low-bleeding-risk cardiac surgery with cardiopulmonary bypass (CPB), but studies in high-bleeding-risk cardiac surgery are limited. The objective of this study is to evaluate the impact of CS on blood coagulation in high-bleeding-risk cardiac surgery with CPB.

**Methods:**

One hundred and ten patients were randomly assigned to either with intra-operative CS group (Group CS) or without intra-operative CS group (Group C). Study endpoints included the incidence of impairment of blood coagulation during perioperative period (peri-op) and the incidence of adverse events during postoperative period (post-op). Peri-op was defined as the period from beginning of anesthesia (anesthesia induction) to 24 h after end of surgery. Post-op was defined as the period from the end of surgery to 24 h after end of surgery. The types of impairment of blood coagulation included heparin residual, coagulopathy due to low PLT, coagulopathy due to low FIB, coagulopathy due to low coagulation factors, hyperfibrinolytic. The sum of above five types was total impairment of blood coagulation. Adverse events included excessive bleeding, resternotomy, etc.

**Results:**

The incidence of heparin residual measured both at the end of surgery and during post-op were significantly higher in Group CS than in Group C (15.09 vs 4.00, 13.21 vs 2.00 %; *p* = 0.024, 0.010, respectively). Similarly, the incidence of total impairment of blood coagulation at the end of surgery and during post-op were significantly higher in Group CS than in Group C (32.08 vs 18.00, 26.42 vs 12.00 %; *p* = 0.043, 0.040, respectively). The incidence of excessive bleeding during post-op was 32.08 % in Group CS compared with 16.00 % in Group C (*p* = 0.038). Intriguingly, CS was associated with a significantly increase in the relative risk ratios for heparin residual and excessive bleeding (*p* = 0.034, 0.049, respectively).

**Conclusions:**

Intra-operative CS could impair blood coagulation in the scenario of high-risk-bleeding cardiac surgery with CPB.

## Background

Individuals who undergo cardiac surgery with cardiopulmonary bypass (CPB) are often exposed to impaired blood coagulation, both as a result of dysfunction of platelet (PLT) and circulatory system was primed of large volumes of fluid [1, 2]. Blood coagulation would be theoretically further impaired when cell salvage (CS) was used in this kind of surgery due to its procedure of centrifugation and washing [3, 4]. However, previous literatures indicated that CS had no impairment on blood coagulation in low-bleeding-risk cardiac surgery with CPB [5, 6].

High- bleeding-risk cardiac surgery with CPB refers to those surgeries with high risk of bleeding due to patients’ poor physical condition and long duration and complexity of the surgery [3, 7, 8]. Among 1.25 million patients undergoing cardiac surgery all over the world each year, a large proportion is with high-bleeding-risk [9].The volume of autologous blood transfusion was much larger in high-bleeding-risk cardiac surgeries with CPB than in low-bleeding-risk ones [9, 10]. Studies demonstrated that volume of autologous blood transfusion was associated with the severity of impairment of blood coagulation [11–13]. We hypothesized that CS could impair blood coagulation when it was used in high-bleeding-risk cardiac surgery.

To date, parameters related to blood coagulation include thromboelastography (TEG) parameters and other hematological parameters such as PLT and FIB. TEG was reported as one of the best indicators of blood coagulation in cardiac surgery with CPB [14–16]. Combination of the test of kaolin-activated sample and the test of heparinase-modified kaolin-activated sample was reported much better to detect heparin residues [17, 18].

**Fig. 1:**
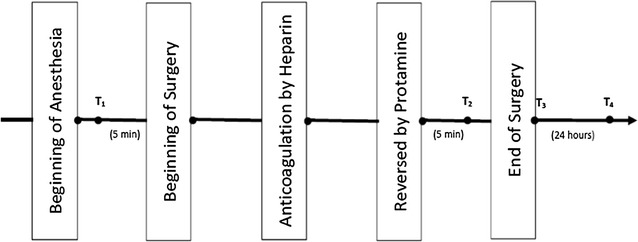
The timeline of events. *T*_*1*_ 5 min before beginning of surgery, *T*_*2*_ 5 min after heparin was reversed by protamine, *T*_*3*_ at the end of surgery, *T*_*4*_ at 24 h after end of surgery. *Perioperative period* (*peri-op*) the period from beginning of anesthesia to 24 h after end of surgery. *Postoperative period* (*post-op*) the period from the end of surgery to 24 h after end of surgery. The period of heparinization: the period from blood anticoagulated by heparin to heparin were reversed by protamine. The period of non-heparinization: the period from beginning of surgery to blood anticoagulated by heparin + the period from heparin were reversed by protamine to end of surgery

Till now, limited studies existed evaluating the impact of CS on blood coagulation in high-bleeding-risk cardiac surgery with CPB. The aim of this prospective randomized and controlled clinical trial was to fill this gap. The primary endpoint of this trial was the incidence of impairment of blood coagulation during perioperative period (peri-op), which was defined as the period from beginning of anesthesia (anesthesia induction) to 24 h after end of surgery (Fig. 1). The secondary endpoint was the incidence of adverse events during postoperative period (post-op), which was defined as the period from the end of surgery to 24 h after end of surgery (Fig. 1).

## Methods

### Patients

This study was approved by the Ethics Committee of the Zhejiang Provincial People’s Hospital (RCT ID: ChiCTR-TRC-13003268). Patients were enrolled in the study after written informed consent was signed. Inclusion and exclusion criteria were shown in Table 1 [3, 7, 8]. One hundred and ten eligible patients were randomly assigned into two groups according to a randomization list: cell salvage group (Group CS) and control group (Group C). Patients in Group CS were treated with intra-operative CS while those in Group C without.

**Table 1:** Inclusion and exclusion criteria of patients

	Inclusion criteria	Exclusion criteria
Opportunity of surgery	Scheduled for cardiac surgery with CPB	Emergency cardiac surgery with CPB
Type of surgery	Multiple valves replacement	The first time single valve replacement
	Bentall	
	Reoperation (at least 1 item above 3 items must be met)	
Characteristics of patient	Age >70 years	
	BSA <1.6 m^2^	
	Cr >15 mg/L	
	Liver Child–Pugh B or C	
	R (TEG) >10 min;	
	HB <13 g/dL (male) or 12 g/dL (female)	
	PLT <50 × 10^9^/L	
	Drug withdrawal <3 days (Aspirin) or 7 days (Clopidogrel) (at least 2 items above 8 items must be met)	

Eligible patient must meet opportunity of surgery and type of surgery and characteristics of patient at the same time

*Multiple valves replacement* aortic valves replacement + mitral valve replacement

*Bentall* aortic valve replacement + ascending aorta and aortic root replacement

*Reoperation* not the first time single or multiple valve replacement

*BSA* body surface area, *TEG* thromboelastography, *HB* hemoglobin, *PLT* platelets, *Cr* creatinine

### CPB

Tranexamic Acid (AMCHA, 30 mg/kg) was infused in all patients after anesthesia induction [9]. The CPB was performed using a HL20 heart–lung machine (MAQUET, Israel) with an OCTOPUS4 extracorporeal membrane oxygenation (Medtronic, USA). The volume of fluid primed was 1.8 L, the main ingredient was ringer’s lactate solution, along with a small amount of albumin and mannitol. Anticoagulation was achieved with heparin to maintain an activated clotting time (ACT) above 480 s. After separation from CPB, heparin was reversed by protamine to a target-ACT of range from 90 to 110 % of base value.

### Autologous blood transfusion

In Group CS, shed blood from wound and mediastina were sucked into the cell saver reservoir (Haemonetics, USA, volume of disposable centrifuge bowl is 125 mL) after anticoagulated by heparin saline during the period of non-heparinization (Fig. 1). The heparinised saline contain 25,000 IU of heparin in 1 L of 0.9 % saline at a rate of 100 mL/h. At the end of CPB, residual blood in the CPB circuit was directly sucked into the reservoir. After being filtrated, centrifugated, washed and concentrated, the recovered blood turned into autologous blood (autologous red blood cell suspension), which was transfused back to the patient immediately. The washing program used in the CS involved a 5:1 ratio between the 0.9 % saline wash and collection of blood from the operating field. All of the autologous blood was transfused back to the patients by the end of surgery. In Group C, shed blood from wound and mediastina during the period of non-heparinization (Fig. 1) were sucked into suction apparatus and were discarded. In Group C, at the end of CPB residual blood in the CPB circuit were sucked into suction apparatus and were discarded. Patients’ bleeding during the period of heparinization (Fig. 1) in two groups were sucked into the CPB circuit.

### Blood transfusion during peri-op

**Fig. 2:**
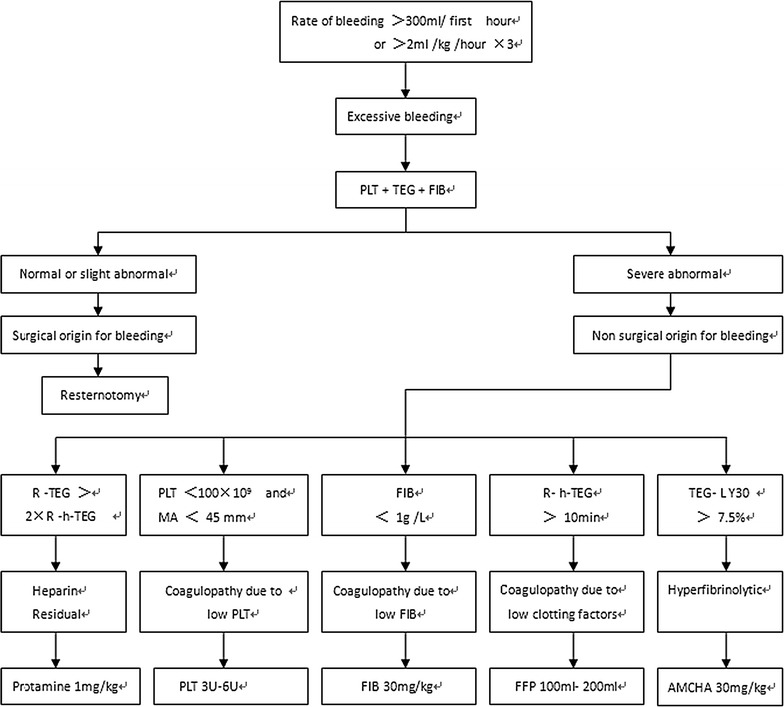
The processing protocol of excessive bleeding during post-op. Once excessive bleeding was diagnosed, patients received treatment based on the results of the tests in the TEG, PLT and FIB. First hour: in the first hour after surgery, hour × 3: for 3 consecutive hours. *TEG* kaolin-activated TEG, *h-TEG* heparinase-modified kaolin-activated TEG, *R* reaction time, *R-TEG* R value in TEG, *R-h-TEG* R value in h-TEG, *MA* maximum amplitude, *LY30* lysis index at 30 min, *PLT* platelets, *FFP* fresh-frozen plasma, *FIB* fibrinogen, *AMCHA* tranexamic acid

After heparin was reversed by protamine, allogeneic red blood cell (RBC) was used if hemoglobin (HB) was lower than 8 g/dL in Group C [3]. In Group CS, allogeneic RBC was used only if HB was still lower than 8 g/dL after transfused all of the autologous blood. Allogeneic fresh-frozen plasma (FFP) and PLT transfusion was used during peri-op according to the protocol (showed in the seventh and eighth line in Fig. 2 [3, 16, 19]. Patients with excessive bleeding during post-op, which was defined as the rate of bleeding greater than 300 mL in the first hour after surgery or greater than 2 mL/kg/h for 3 consecutive hours, were treated according to the same protocol (Fig. 2).

### Study variables

Basal and operative characteristics: including age, gender, BSA, type of surgery, complications, CPB time, surgical time, tracheal intubation time, length of ICU stay, length of hospital stay, the volume of residual blood in CPB circuit, the volume of intra-operative blood loss, the volume of mediastina tube drainage (MTD) in 6 and 24 h after surgery, intra-operative dosage of heparin and protamine. The volume of intra-operative blood loss was calculated as the volume sucked into cell saver reservoir minus the volume of heparin saline for anticoagulation in Group CS or the volume sucked into suction apparatus in Group C.

Volume of blood transfusion during peri-op: including the volume of autogenous blood in Group CS, the volume of allogeneic blood (RBC, PLT and FFP) in two groups.

Hematological parameters during peri-op: including general parameters and parameters related to blood coagulation. General parameters included RBC and HB. Parameters related to blood coagulation included TEG parameters (Haemonetics, USA), PLT and FIB. TEG parameters included R value (R-TEG and R-h-TEG), MA and LY 30. R (reaction time, normal range from 10 to 19 min) represents the time to initiation of clot formation. R-TEG and R-h-TEG are the R value of test of kaolin-activated sample and heparinase-modified kaolin-activated sample, respectively; MA (maximum amplitude, normal range from 54.5 to 72.5 mm) represents the maximum clot strength; LY30 (lysis rate-30, normal range from 0 to 7.5 %) represents the rate of cloth lysis 30 min after MA. Venous blood was tested at the following four time points or anytime when excessive bleeding occurred during post-op: at the time of after anesthesia induction and 5 min before beginning of surgery (T_1_), 5 min after heparin was reversed by protamine during surgery (T_2_), at the end of surgery (T_3_) and at 24 h after end of surgery (T_4_) (Fig. 1).

Impairment of blood coagulation during peri-op: impairment of blood coagulation was diagnosed according to the test results of hematological parameters related to blood coagulation (showed in the seventh and eighth line in Fig. 2). Impairment of blood coagulation were classified into five types: heparin residual, coagulopathy due to low PLT (decreasing of PLT results in hypocoagulability), coagulopathy due to low FIB (shortage of FIB results in hypocoagulability), coagulopathy due to low coagulation factors (shortage of other coagulation factors except for factor results in hypocoagulability) and hyperfibrinolytic (showed in the seventh and eighth line in Fig. 2) [17, 19]. The sum of above five types was total impairment of blood coagulation.

Adverse events during post-op: including excessive bleeding, resternotomy, cardiovascular failure, severe arrhythmias, myocardial infarction, infection, renal failure, respiratory failure, epileptic syndrome, cognitive decline and death.

### Statistical analysis

Data are presented as mean (standard deviation, SD) for continuous parametric values, or as numbers with percentage for categorical data. For continuous data, the two-sample two-sided Student’s t test or the Wilcoxon Rank-Sum two-sample two-sided test was used. For categorical data, the *χ*^*2*^ test or Fisher’s exact-test was used. Considering some variables may influence relevant clinical outcomes potentially, the logistic regression model was used to analyze the outcomes in Table 4. Odds ratios (ORs) resulting from logistic regression analysis were used to estimate relative risk (RR) ratios. Data analysis was performed using SAS statistical software (version 9.1; SAS Institute Inc., Cary, NC, USA). *P* value of less than 0.05 was considered as statistically significant.

## Results

### Basal and operative characteristics

**Fig. 3:**
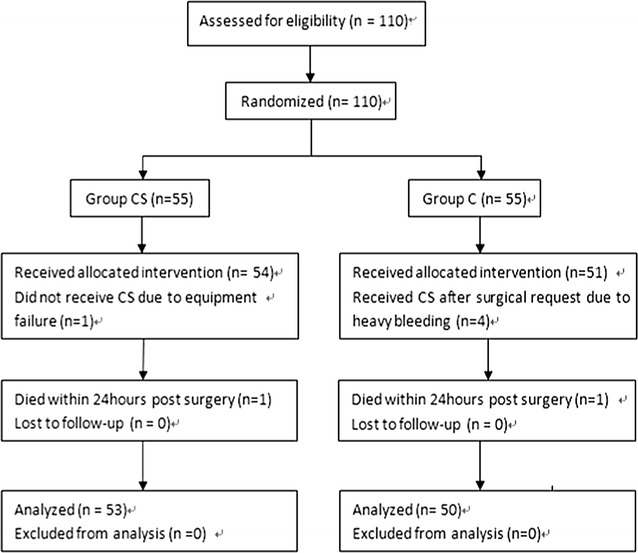
CONSORT diagram of flow of patients

Of the total 110 patients randomized between April 2013 and September 2014, 55 were allocated to Group CS and 55 to Group C (Fig. 3). There was no difference between two groups in the listed variables (Table 2).

**Table 2:** Patients’ basal and operative characteristics

Variable	Group CS (n = 53)	Group C (n = 50)	P value
Age (years)	50.42 (15.43)	52.53 (15.65)	0.549
>70	12 (22.64)	13 (26.00)	0.691
Male	27 (50.94)	24 (48.00)	0.765
BSA	1.73 (0.14)	1.73 (0.13)	0.946
<1.6 m^2^	5 (9.43)	4 (8.00)	1.000
Surgery type			0.922
Multiple valve	36 (67.92)	33 (66.00)	
Bentall	8 (15.09)	7 (20.00)	
Reoperations	9 (16.98)	10 (14.00)	
Preoperative complication			
Renal dysfunction	19 (35.84)	18 (36.00)	0.987
Liver insufficiency	9 (16.98)	7 (14.00)	0.676
Coagulation disorders	15 (28.30)	13 (26.00)	0.793
HB levels lower (<13 g/dL in male or <12 g/dL in female)	21 (39.62)	23 (46.00)	0.513
	17 (32.08)	18 (36.00)	0.674
PLT levels lower (<50 × 10^9^/L) intake of aspirin or clopidogrel	12 (22.53)	11 (22.00)	0.938
CPB time (min)	136.05 (36.82)	126.65 (25.63)	0.342
Surgical time (min)	302.56 (28.02)	295.94 (22.02)	0.220
Tracheal intubation time (hour)	13.92 (10.83)	13.15 (3.62)	0.434
Length of ICU stay (hour)	19.42 (4.43)	20.42 (4.16)	0.099
Length of hospital stay (day)	22.56 (4.23)	23.38 (4.54)	0.206
Residual blood in CPB circuit	853.67 (50.46)	837.22 (92.53)	0.200
Intra-operative blood loss (mL)	1640.96 (87.73)	1609.03 (137.91)	0.072
MTD (mL)			
In 6 h	333.02 (147.14)	295.03 (152.82)	0.301
In 24 h	657.78 (272.94)	591.96 (171.53)	0.071
Intra-operative heparin dose (mg)	244.63 (49.82)	241.06 (49.35)	0.778
Intra-operative protamine dose (mg)	428.37 (92.83)	417.36 (92.02)	0.478

Data are presented as mean (SD) or number (percentage)

*MTD* mediastinal tube drainage, patients with resternotomy were not calculated

### Blood transfusion during peri-op

The mean volume of autologous blood transfusion in Group CS was 4.13 U (0.31 U). The proportion of allogeneic RBC transfusion during peri-op was 41.51 % in Group CS versus 78.00 % in Group C. The mean volume of allogeneic RBC transfusion during peri-op was 2.66 U in Group CS versus 5.40 U in Group C. Both the proportion and volume of allogeneic RBC transfusion during peri-op were significantly lower in Group CS than in Group C (Table 3).

**Table 3:** Allogeneic blood transfusion during peri-op

Variable	Group CS (n = 53)	Group C (n = 50)	P value
RBC			
Proportion	22 (41.51)	39 (78.00)	0.0002
Volume (U)	2.11 (2.66)	5.40 (3.48)	<0.0001
FFP			
Proportion	10 (18.87)	9 (18.00)	0.910
Volume (mL)	118.76 (253.82)	129.00 (284.92)	0.953
PLT			
Proportion	12 (22.64)	10 (20.00)	0.744
Volume (U)	1.81 (3.56)	1.92 (3.94)	0.916

Data are presented as mean (SD) or number (percentage)

### Impairment of blood coagulation during peri-op

**Fig. 4:**
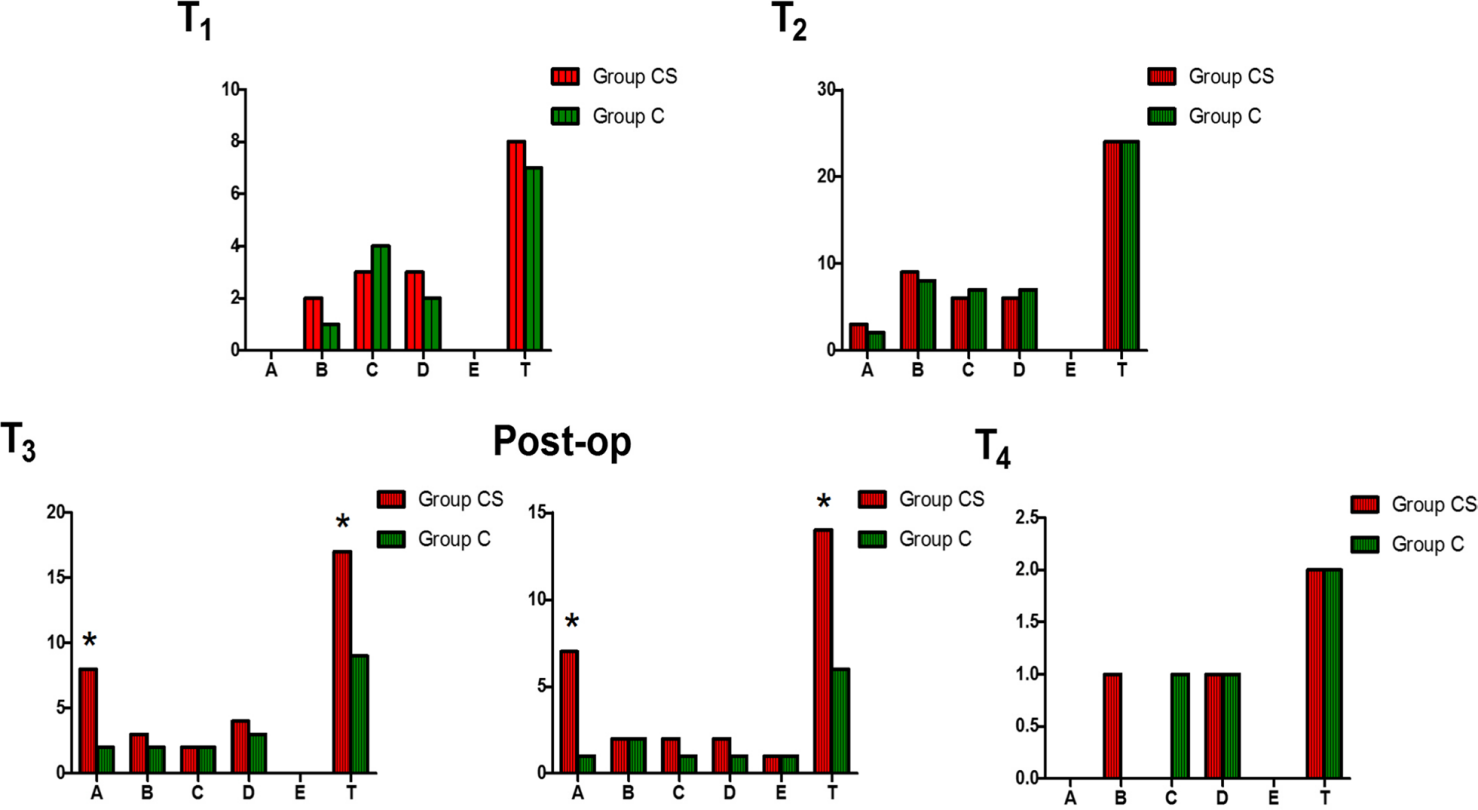
Comparison of impairment of blood coagulation during peri-op between two groups. *x-axis* type of impairment of blood coagulation, *A* heparin residual, *B* coagulopathy for low PLT, *C* coagulopathy for low FIB, *D* coagulopathy for low clotting factors, *E* hyperfibrinolysis, *T* total (A + B + C + D + E). *y-axis* cases of each type, *T*_*1*_ at the time of after anesthesia induction and 5 min before surgery, *T*_*2*_ 5 min after heparin was reversed by protamine during surgery, *T*_*3*_ at the end of surgery, *post-op* during postoperative period, *T*_*4*_ at the time of 24 h after surgery. *p = 0.024(T_3_ − A), 0.043(T_3_ − T), 0.010 (post-op − A), 0.040 (post-op − T), respectively

The incidences of heparin residual at T_3_ and during post-op were significant higher in Group CS (15.09 and 13.21 %) than in Group C (4.00 and 2.00 %), with *p* value as 0.024 and 0.010, respectively (Fig. 4). Similarly, the incidences of total impairment of blood coagulation at T_3_ and during post-op were significant higher in Group CS (32.08 and 26.42 %) than in Group C (18.00 and 12.00 %), with *p* value as 0.043 and 0.040, respectively (Fig. 4). The RR value of CS with heparin residual was 6.10. CS was associated with an increase in the RR for heparin residual (*p* = 0.034) (Table 5).

### Adverse events during post-op

The incidence of excessive bleeding during post-op were significantly higher in Group CS (32.08 %) than in Group C (16.00 %) (Table 4). The RR value of CS with excessive bleeding was 4.58. CS was associated with an increase in the RR for excessive bleeding (*p* = 0.049) (Table 5).

**Table 4:** Relationship of CS with impairment of blood coagulation and adverse events

Variable	RR	95 % CI	P value
Heparin residual	6.10	1.14–32.60	0.034
Coagulopathy for low PLT	0.61	0.14–2.61	0.509
Coagulopathy for low FIB	1.35	0.40–4.48	0.629
Coagulopathy for low clotting factors	0.56	0.09–3.30	0.521
Excessive bleeding	4.58	1.02–18.34	0.049
Myocardial infarction	0.15	0.01–1.99	0.150
Cognitive decline	<0.001	<0.001–999.99	0.938

*RR* relative risk, *CI* confidence interval

**Table 5:** Summary of adverse events during post-op

Variable	Group CS (n = 53)	Group C (n = 50)	P value
Excessive bleeding	17 (32.08)	8 (16.00)	0.038
Resternotomy	3 (5.66)	2 (4.00)	1.000
Cardiovascular failure	6 (11.32)	7 (14.00)	0.682
Severe arrhythmias requiring treatment	4 (7.55)	3 (6.00)	1.000
Myocardial infarction	1 (1.89)	4 (8.00)	0.196
Infection	7 (13.21)	6 (12.00)	0.948
Wound	3 (5.66)	2 (4.00)	1.000
Others	4 (7.55)	4 (8.00)	1.000
Renal failure	5 (9.43)	4 (8.00)	1.000
Respiratory failure	3 (5.66)	2 (4.00)	1.000
Epileptic syndrome	1 (1.89)	1 (2.00)	1.000
Cognitive decline	0 (0.00)	3 (6.00)	0.111
Death	0 (0.00)	0 (0.00)	

Data are presented as number (percentage)

## Discussion

In our present study, significant differences of the incidence of heparin residues and total impairment of blood coagulation during peri-op were found between two groups. We also found that the incidence of excessive bleeding during post-op was significantly higher in group CS than in group C. In addition, we found that CS was a dependent risk factor for heparin residues and excessive bleeding. These results indicate that intra-operative CS could impair blood coagulation in the scenario of high-risk-bleeding cardiac surgery with CPB, which is consistent with our hypothesis.

CPB determines an activation of the coagulation system [1, 2]. CS system determines a further activation of the coagulation system [3, 4, 20]. The enhanced activation of the coagulation system induced a strong thrombin generation. Thrombin generation directly consumes coagulation factors, activates PLT, promotes fibrinolysis, lowers fibrinolysis inhibition and stimulates the release of tissue plasminogen activator from endothelial cells [20]. These conditions were considered as a possible trigger for a consumption coagulopathy and hyperfibrinolysis.

In our practice, processing of recovered blood during the CS process involves the collection of blood and mixing with heparinised saline, centrifugation of the cells, followed by saline washing prior to re-transfusion to the patient. The washing program used in the CS involved a 5:1 ratio between the 0.9 % saline wash and collection of blood. This process removes plasma proteins and coagulation factors. In addition, hemodilution during CPB and the fluid therapy during post-CPB may result in dilutional coagulopathy [12, 21].

To prevent thrombogenesis during the collection of blood, as the standard protocol practiced in our study, the heparinised saline containing 25,000 IU of heparin in 1 L of 0.9 % saline at a rate of 100 mL/h was used. Previous studies revealed that after the recovered blood processed by the CS system. The autologous blood may contain heparin about 500 IU/L [12]. In our study, the incidence of heparin residual at the end of surgery were 15.09 % in Group CS and 4 % in Group C, respectively. This result reveals that residual heparin from the CS system could worsen the coagulopathy and also is an important trigger for the impairment of blood coagulation in these patients.

The dose of heparin in the autologous blood that was transfused back to the patient could vary depends on the transfusion volume, concentration and drip rate of heparinised saline. We found it is very cumbersome in clinical practice to estimate the level of heparin in the autologous blood. Anticoagulation was commonly monitored with ACT. Clinicians would have to use protamine to reverse the residual heparin according to the ACT value. The value of ACT may be prolonged by hemodilution which is very common in this kind of surgery [22]. Thus, ACT is a partially reliable bedside method for monitoring the adequacy of anticoagulation. Recent studies revealed that the TEG is a more reliable and sensitive method for assessment of the heparinization level after the autologous blood transfusion [16, 23]. In our study, the R values of heparinase-modified kaolin-activated samples (R-h-TEG) were compared with those of kaolin-activated samples (R-TEG) to assess heparin residual.

The appropriate concentration of the anticoagulant heparin saline and the degree of washing performed by the CS are vital to preventing the heparin residual from the autologous blood. The concentration of the anticoagulant heparin saline used was 50,000 IU/L, even 30,000 IU/L in some report [24]. In our study, although the concentration 25,000 IU/L and the degree of washing 5:1 volume ratio between the 0.9 % saline wash and blood were used, the incidence of heparin residual at the end of surgery in Group CS was up to 15.09 %. This implies that the concentration of 25,000 IU/L may be still risky [22]. The solution to this specific problem may be either reducing the concentration of anticoagulant heparin saline or administering an appropriate dose of protamine to reverse heparin depends on the monitoring of TEG. However, reducing the concentration of heparin may result in thrombogenesis during the collection of blood from the operating field. On the other hand, increasing the dose of protamine may cause adverse events such as allergic reactions. Future studies with a large sample size are urgently begged to further evaluate the appropriate concentration of the anticoagulant heparin saline.

Literatures indicated that intra-operative CS has no impairment on blood coagulation in low-bleeding-risk cardiac surgery with CPB [5, 6]. In our present study, intra-operative CS could impair blood coagulation in the scenario of high-risk-bleeding cardiac surgery with CPB. Previous studies reported that the volume of autologous blood transfused back to the patient in high-bleeding-risk cardiac surgery with CPB was much larger than in low-bleeding-risk cardiac surgery [9, 10]. All types of impairment of blood coagulation are associated with the volume of autologous blood. The more autologous blood transfused, the more severe the impairment of CS on blood coagulation to be expected.

To the best of our knowledge, our study is the first prospective randomized controlled clinical trial investigating the impact of CS on blood coagulation in high-risk-bleeding cardiac surgery with CPB. As the increasing popularity of the CS, more attention should be focused on the patients’ blood coagulation after the autologous blood transfusion, especially in the patients in high-bleeding-risk cardiac surgery with CPB. This study highlighted the importance of the blood coagulation monitoring which could be overlooked in these situations [24]. We strongly recommend application of monitoring of blood coagulation by the TEG combined with kaolin-activated sample and heparinase-modified kaolin-activated sample. Our experience was valuable for anesthesiologists and surgeons to deal with the impairment of blood coagulation in this kind of surgery.

One limitation of our study was the evaluation standard of high-bleeding-risk cardiac surgery. Because there is no universal standard so far, the standard we employed may need some modification. In addition, double blind research method was not adopted in the present study due to objective reasons, which may include some bias. This protocol may affect our results. Another limitation of present study was the administration of AMCHA which lowers fibrinolysis. Accordingly, the incidence of hyperfibrinolysis in two groups were lower than usual.

## Conclusion

Our preliminary data support a proposal that intra-operative CS could impair blood coagulation in the scenario of high-risk-bleeding cardiac surgery with CPB.

## Abbreviations

CS: cell salvage
CPB: cardiopulmonary bypass
Peri-op: perioperative period, the period from anesthesia induction to 24 h after surgery
Post-op: postoperative period, the period from the end of surgery to 24 h after surgery
TEG: thromboelastography, kaolin-activated TEG
h-TEG: heparinase-modified kaolin-activated TEG
R: reaction time
R-TEG: R value in TEG
R-h-TEG: R value in h-TEG
MA: maximum amplitude
LY30: lysis index at 30 min
AMCHA: tranexamic acid
ACT: activated clotting time
RBC: red blood cell
HB: hemoglobin
PLT: platelets
FIB: fibrinogen
FFP: fresh-frozen plasma
MTD: the volume of mediastina tube drainage
RR: relative risk
